# Supplementary material for: Limitations of Spatial Judgment Bias Test Application in Horses (Equus ferus caballus)
Source: Animals (Basel). 2022 Nov 3;12(21):3014. doi: 10.3390/ani12213014 (PMC9654000; doi:10.3390/ani12213014)
Supplement: Supplementary file 1 [file animals-12-03014-s001.zip › animals-1964481-supplementary.pdf]

animals MDPI

Article

Limitations of Spatial Judgment Bias Test Application in Horses (*Equus Ferus Caballus*)

Giovanna Marliani *, Irene Vannucchi , Irini Kiumurgis and Pier Attilio Accorsi

Department of Veterinary Medical Sciences, University of Bologna, Via Tolara di Sopra, 50, Ozzano dell'Emilia (BO) 40064 Italy; irene.vannucchi@studio.unibo.it (I.V.); irini.kiumurgis@studio.unibo.it (I.K.); pierattilio.accorsi@unibo.it (P.A.A.)
* Correspondence: giovanna.marliani2@unibo.it; Tel.: +393932693823

Table S1. Ethogram used to assess horses' stress during JBT test [32-37].

Stress-related behaviours	
**Neck Turn**	The horse turns its head and neck from the side opposite to the stimulus to which it is relating. During this movement, the trunk and limbs are still. Both ears are facing forward, or one ear is facing forward and the other is towards the stimulus.
**Head Turn**	The horse turns its head left or right away from the stimulus independently from its handler.
**Vacuum Chewing/Licking and Chewing**	The horse moves its mouth and chews, and sometimes it protrudes its tongue (licking and chewing), without any stimulus in its mouth.
**Neck Shake**	Rapid rhythmic rotation of the head and neck
**Body Shake**	Rapid rhythmic rotation of the entire body
**Yawning**	Deep long inhalation with mouth widely open and jaws either directly opposed or moved from side to side
**Head toss**	The horse makes an oscillation or rotational movement with the head. It can be emitted from a standstill position or during locomotion.
**High Tail**	Tail is raised with its fleshy part horizontal or above horizontal.
**Clenched Tail**	The tail is pressed under the region inguinal or between the hind limbs.
**Tail Swishing**	Quick and lateral movements of the tail.
**Ears moving or scanning**	The ears are continuously moved back and forth at varying speeds and sometimes one ears points forward and the other backward.
**Ears Pinned Back**	Ears pressed caudally against the head and neck.
**Head Raised**	The horse extends slightly its neck, helds the head higher than the normal carriage, and the nose is extended upward
**Defecation**	Expelling of feces
**Rolling**	The horse lyes on the ground and roll side to side, rubbing its back on the ground
**Self-mutilation**	Bite movement directed at own body usually the flanks or the chest and limbs (3)

**Scratching**	Rubbing part of the body against surface of object, fance or stable, using also mouth and hind limb.
**Pawing**	Movement of one front limb back and forth on the ground or horizontal surface
**Sniffing**	Exploratory behaviour towards the floor or an object
**Liking Object**	Exploratory behaviour with tongue towards and object
